# Association of physical activity with incident dementia and cognitive decline among Australian older adults

**DOI:** 10.1007/s11357-026-02179-x

**Published:** 2026-03-04

**Authors:** Yang Chen, Danijela Gasevic, Alice Owen, Shivangi Shah, Dragan Ilic, Joanne Ryan, Robyn L. Woods, Stella Talic, Rory Wolfe, Suzanne G. Orchard

**Affiliations:** 1 School of Public Health and Preventive Medicine, Monash University, 553 St Kilda Road, Melbourne, Victoria 3004 Australia; 2 Baker Heart and Diabetes Institute, 75 Commercial Rd, Melbourne, VIC 3004 Australia; 3 Centre for Global Health, The Usher Institute, The University of Edinburgh, Teviot Place, Edinburgh, EH8 9AG UK

**Keywords:** Ageing, Physical activity, Older adults, Gerontology, Dementia, Cognitive decline

## Abstract

We investigated the association between physical activity (PA) intensity and dementia and cognitive decline in Australian community-dwelling older adults. This prospective cohort study uses data from 11,655 older adults (mean age (standard deviation, SD) = 75.0 (4.2) years, 53.4% females, 52.9% had completed ≥ 12 years of education), who were participants in the ASPirin in Reducing Events in the Elderly (ASPREE) clinical trial and its observational follow-on (ASPREE-XT) and the ASPREE Longitudinal Study of Older Persons (ALSOP) sub-study. Participants self-reported the exposure of usual PA intensity (never/rarely, light, moderate, vigorous). Dementia was adjudicated using the Diagnostic Manual of Mental Disorders 4th Edition and supporting evidence; cognitive decline was defined as a > 1.5 standard deviation reduction from baseline on any cognitive assessment. Associations were explored using hazard ratios from Cox regression models with adjustment for demographic, socio-economic, behavioural and clinical factors. Of 11,655 participants (mean age 75.0 years) over a median 10.4-year follow-up, 931 (8.0%) developed dementia and 3552 (30.8%) cognitive decline. Compared with light PA, dementia or cognitive decline risk was 79% (95% CI 1.14–2.83) and 58% (95% CI 1.21–2.05) higher, respectively, among those who rarely/never engaged in PA, but no further decreases occurred with higher levels of PA. Engagement in light PA may help lower dementia and cognitive decline risk in older adults.

**Graphical abstract:**

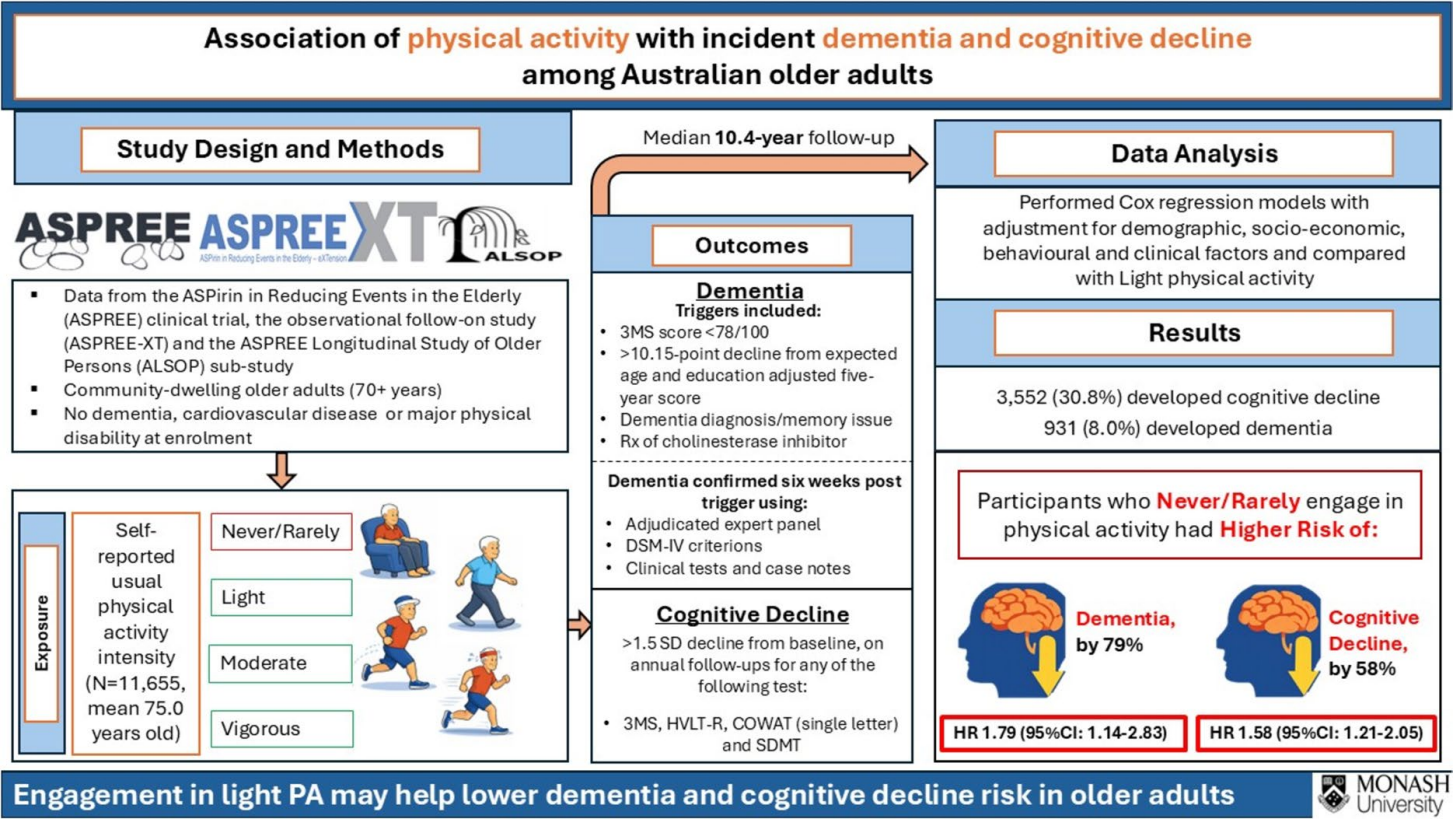

**Supplementary information:**

The online version contains supplementary material available at 10.1007/s11357-026-02179-x.

## Introduction

As the global population ages, the prevalence of age-related comorbidities continues to rise [1]. Cognitive decline and dementia are among the leading causes of dependency and disability in older adults [1].The risk of developing dementia increases significantly with age, from 5% among individuals aged 71–79 to 37.4% in those aged 90 and above [2]. In 2023, an estimated 55 million people worldwide were living with dementia, a figure projected to triple and reach 152 million by 2050 [1].

Physical activity (PA) is a modifiable risk factor that may potentially delay or prevent the onset of dementia [3, 4]. Meeting PA recommendations in mid-life can reduce the risk of developing dementia, and it is thought that later life PA may also be important for risk reduction [5]. The World Health Organization currently recommends engaging in at least 150 min of moderate or 75 min of vigorous PA and engagement in muscle-strengthening activities on at least 2 days per week [6]. Yet, for those who are unable to engage at the recommended PA levels, notably older individuals, even small amounts of PA, such as taking the stairs instead of the elevator, or walking for short periods throughout the day, can accumulatively add to the daily PA total and may contribute to reduced cognitive decline and improved memory and brain health [7]. However, whether the intensity of habitual PA in later life is a driver or modifier of risk is unclear [3, 4].

Most studies to date have assessed PA engagement using dichotomous outcomes (yes vs no, high vs low or moderate-to-vigorous vs light), limiting the granularity of intensity-specific insights [3, 4]. A large systematic review published by Iso-Markku et al. (2021) involving 58 studies synthesised that engaging in any PA was found to decrease the risk of developing dementia by 20% compared to no PA engagement (pooled RR, 0.80; 95% CI, 0.77–0.84) [3]. Similarly, another systematic review including 21 studies reported that high PA intensity engagement decreased the risk of cognitive decline by 35% (RR, 0.65; 95% CI, 0.55–0.76) and dementia by 14% (RR, 0.86; 95% CI, 0.76–0.97), compared to those who only engage in low intensity PA [4].

To date, only a limited number of studies have stratified PA by intensity among community-dwelling older adults, and even fewer have investigated whether light-intensity PA is beneficial for the prevention of dementia [3, 4]. Light PA may be particularly important for older adults, as not everyone has the physical capacity to engage in higher-intensity activity or to fully benefit from its potential preventive effects against dementia [3, 4]. Among the limited evidence examining associations between stratified PA intensities (including light PA) and cognitive outcomes, studies have certain limitations: the study population was specific to certain countries, such as Spain, where patterns of PA engagement may be different to Australia due to cultural and environmental differences [8], studies included both middle-aged and older adults without stratifying by age [9], or found a U-shaped association between PA intensity and risk of cognitive performance [10] or that maximal PA intensity may result in increased cognitive decline [11]. A Mendelian randomisation study using genetically predicted proxies for PA found that genetically predicted moderate-to-vigorous PA reduced cognitive performance, suggesting a potential adverse effect of higher intensity PA on cognition [10]. Similarly, in a study conducted by Roy et al., maximal intensity of cardiopulmonary exercise testing among children revealed that maximal PA intensity may reduce cognitive performance [11]. These findings challenge the assumption that higher PA intensities provide higher protection against dementia and cognitive decline and highlight the need for further research examining PA intensity. A better understanding of the impact of PA intensities on cognition is critical for informing health promotion strategies. This is especially important given that 27.5% of the global older adult population do not currently meet WHO’s recommended PA intensity guidelines for optimal health benefits, and that increasing age in later life may be perceived as a barrier to PA participation [12].

This study explores the association between various intensities of PA undertaken in later life and cognitive decline and dementia in a large cohort of Australian community-dwelling older adults, using validated cognitive assessment tools.

## Methods

This is a secondary analysis using data from the ASPirin in Reducing Events in the Elderly (ASPREE) clinical trial [13], its observational follow-on cohort study (ASPREE-XT) [14] and the ASPREE Longitudinal Study of Older Persons (ALSOP) sub-study [15].

The ASPREE clinical trial was a randomised placebo-controlled trial of daily low-dose aspirin (100 mg) in initially healthy, community-dwelling adults aged 70 years or older (or 65 years or older in the United States of America (US) minority groups) conducted in Australia and the US [13]. Participant eligibility characteristics of the ASPREE clinical trial have been previously published [13]. Of note, participants with a Modified Mini-Mental State (3MS) examination (for global cognition) score of < 78/100 or with a self-report/physician diagnosis of dementia were excluded [13]. The ASPREE clinical trial concluded on the 12th of June, 2017 (median follow-up period of 4.7 years), and surviving participants were invited to participate in the ASPREE-XT follow-on study [14].

Australian participants in the ASPREE study were invited to join the ALSOP sub-study within their first year of enrolment, which was designed to investigate a wide array of general, behavioural, social, economic and environmental factors that may influence future health and well-being among adults aged 70 years and older [15].

### Exposure assessment—physical activity (PA)

PA intensity was assessed at the ALSOP baseline social questionnaire, with participants asked to self-report their usual intensity of PA behaviour in a typical week via the following question: "*Thinking about how much physical activity you do at present, in a typical week, which of the following best describes your level of activity*?" Response options, based upon the WHO standards for exercise in older adults, and the categories used in this analysis, included: rarely/never engaged in PA, light PA (reference group for analyses), moderate PA, and vigorous PA [15].

### Outcome assessment—dementia and cognitive impairment

Dementia was determined as a clinically adjudicated secondary endpoint during the ASPREE study [16]. Triggers for dementia were:

- a score of < 78/100 on the 3MS, or
- a decline of > 10.15 points from the expected age and education adjusted five-year score,
- a medical diagnosis of dementia or memory problems, or
- a prescription for a cholinesterase inhibitor.

Six weeks after participants triggered for dementia, they attended a cognitive assessment visit (to avoid potential impacts of temporary delirium brought on by non-dementia-related conditions) for additional cognitive tests. The outcomes of the cognitive assessment visit, along with clinical case notes, brain CT/MRI, laboratory tests or dementia screening blood tests, were presented as supplementary supporting evidence and reviewed against the Diagnostic Manual of Mental Disorders, Fourth Edition (DSM-IV) criteria for dementia, by a blinded independent panel of specialists [17]. The cognitive assessment included the Alzheimer Disease Assessment Scale-Cognitive Subscale [18], the Color Trials [19], Lurian overlapping figures [20] and, to ascertain if cognitive impairment led to social/occupational functional loss, the Alzheimer Disease Cooperative Study Activities of Daily Living Scale [21].

Cognitive function was assessed as a continuous variable at baseline and years one, three, five, seven and at the close-out visit (2017) of the ASPREE clinical trial, and annually during the ASPREE-XT study. Cognitive decline (no dementia) was defined as > 1.5 standard deviation (SD) decline in cognitive scores from baseline on any of the study visit cognitive battery assessments: including the 3MS for global cognition [22], the Hopkins Verbal Learning Test–Revised Delayed Recall (HVLT-R) task for episodic memory [23], the single letter (F) Controlled Oral Word Association Test (COWAT) for language and executive function [24] and the Symbol Digit Modalities Test (SDMT) for psychomotor speed [25].

### Covariates

Baseline covariates were selected based on previous literature [3, 4, 8–11], and included self-reported age (years), sex (male, female), education (< 12 years, ≥ 12 years), smoking and alcohol status (current, former, never), living status (lives alone, does not live alone), area-level socioeconomic status (validated measure; Index of Relative Socio-economic Advantage and Disadvantage (IRSAD)) and annual income status (< AUD$20,000, $20,000–$49,999, $50,000–$99,999, ≥ $100,000, or prefer not to say).

IRSAD was determined from the 2011 Australian Census of Population and Housing [26]. Participants were stratified into quintiles, where the lowest quintile (Q1) indicated an area with greatest disadvantage, while the highest quintile (Q5) indicated an area with least disadvantage [26].

Additional variables included baseline information on body mass index (continuous), diabetes mellitus (yes/no), hypertension (yes/no), depression (yes/no), frailty (non-frail and pre-frail/frail) and dyslipidaemia (yes/no). Apolipoprotein E (APOE4) allele (hetero- and homozygous) carriers (yes/no) and PA during middle-age were only used in sensitivity analyses.

Baseline diabetes mellitus was defined as a fasting blood glucose level ≥ 126 mg/dL, antihyperglycemic medication use or self-reported diagnosis of diabetes. Hypertension was defined as the presence of high blood pressure (systolic ≥ 140 mmHg or diastolic ≥ 90 mmHg) or use of antihypertensive medications. Depression symptoms were assessed through the Center for Epidemiological Studies Depression (CESD-10) scale [27]. A score of > 8 (out of 30) on the CESD-10 scale was defined as living with depressive symptoms [27]. Dyslipidaemia was defined as statin use at baseline or presence of an elevated total serum cholesterol of ≥ 212 mg/dL (≥ 5.5 mmol/L) or LDL ≥ 160 mg/dL (> 4.1 mmol/L) (levels deemed to represent dyslipidaemia classifications in both Australian and US older adults) [28, 29]. Frailty was assessed using a modified Fried frailty phenotype score [30]. Five components were used to determine Fried frailty, including slow gait speed, low grip strength, low BMI categories, low physical activity (participants were considered to have low activity if they responded “no” or “less than 10 min” to the questions “In the past 2 weeks, have you done any walking outside the home?” and “What is the longest amount of time you walked without sitting down?”, ensuring this measure did not overlap with the outcome of interest for this study) and exhaustion ((evaluated in  the CESD-10) [28]. The low physical activity component of frailty captured extreme inactivity, whereas PA intensity was assessed independently using World Health Organization–based categories of usual activity intensity. These distinct operational definitions reduce conceptual overlap between frailty and the PA exposure. Participants with ≥ 3 components of frailty were classified as frail, 1–2 components as pre-frail and none as non-frail. Further details have been previously published [30]. Due to the small number of participants classified as frail, the frail and pre-frail categories were combined for statistical analyses.

The participant’s usual intensity of PA during middle-age was self-reported by asking the question: “Thinking back to when you were middle-aged (40–55 years old), in a typical week, what best describes your level of PA”. Participants were able to choose from the same WHO standard PA intensities mentioned above.

### Data analysis

**Fig. 1:**
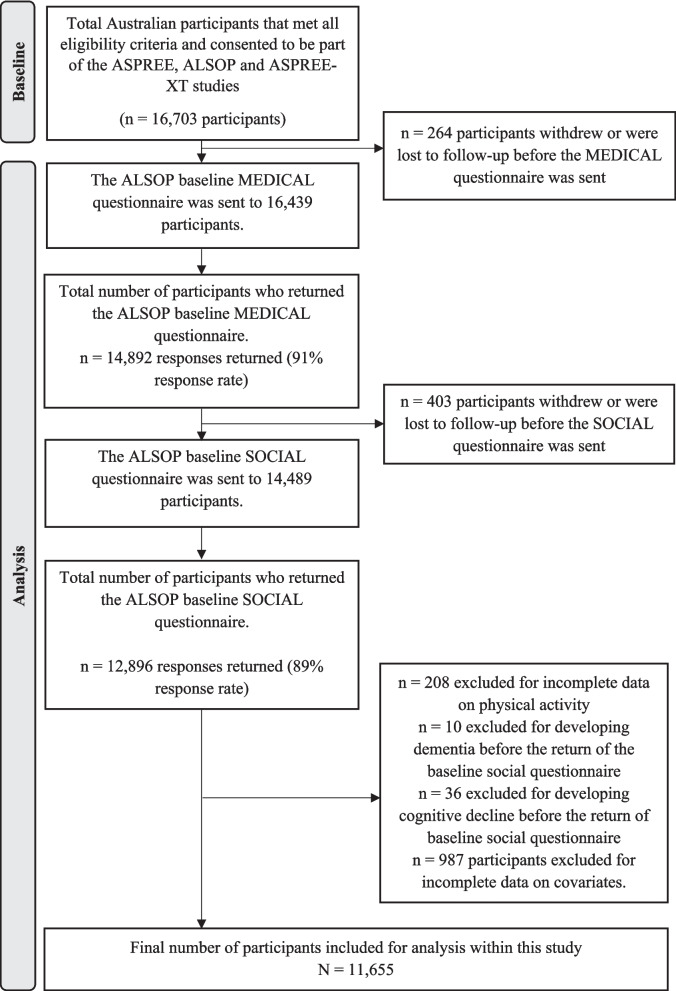
Participant inclusion flow chart

Study participants who had incomplete data on any variables of interest or did not complete an ALSOP social questionnaire were excluded from the analysis (Fig. 1). Since the majority of participants received and returned the baseline ALSOP questionnaires between 3 and 6 months after ASPREE randomisation (29.5% by 3 months and 72.6% by 6 months), the ASPREE trial baseline was used as baseline (time zero) for this analysis (Supplementary Figure S1). However, to avoid immortal time bias, all participants who developed dementia or cognitive decline before the baseline ALSOP questionnaire (from which the PA data was obtained) was returned were also excluded from the analysis. After all ALSOP questionnaires were returned, the study period commenced.

Baseline participant characteristics were stratified by categories of PA intensity and presented as counts (n) and percentages (%) for categorical data. Continuous variables such as age, which were not skewed based on the results of a Q-Q plot, were presented using mean and SD. The association of PA intensity with dementia or cognitive decline were explored using Cox proportional hazards (PH) models. After graphical assessment of the Schoenfeld residuals test, the Cox PH assumptions were met for dementia (*p* = 0.11) and cognitive decline (*p* = 0.19). Model one adjusted for age, sex, education, annual household income, living arrangements, IRSAD, smoking status and alcohol consumption. Model two was model one with additional adjustment for baseline BMI, diabetes mellitus, hypertension, depression, frailty and dyslipidaemia. Hazard ratios (HR) with 95% confidence intervals (95% CI) were reported. Due to a small proportion of participants in the never/rarely PA group (*n* = 152), the light PA group was used as the reference category.

To explore potential effect modifications [3, 4, 8–11], we performed an interaction analysis of PA intensity with age, sex and income. None modified the association of PA intensity with dementia or cognitive decline (p-value > 0.88 for all); thus, all subsequent analyses were performed on the whole sample. A spearman correlation matrix was performed to describe correlations among the covariates and with PA to rule out multicollinearity. The highest correlation coefficient was 0.26 (living status and sex), indicating no multicollinearity in the data (Supplementary Figure S2).

To explore the robustness of our findings, sensitivity analyses were performed: (1) the first used the never/rarely PA group as the reference group to test if any PA (categorised as anyone engaging in light/moderate/vigorous PA) intensity was beneficial; (2) excluded participants who developed dementia or cognitive decline within one year of study commencement, to address potential of reverse causality bias; (3) included additional adjustments for self-reported PA during middle-age and APOE4 allele status, since lifelong engagement in PA and genetic factors may affect cognitive health [3, 4, 8–11]; and (4) involved multiple imputation to account for participants with missing information about covariates and exposures. We combined results across imputed datasets using Rubin’s rules and compared the results with the complete-case analysis (Supplementary Methods).

All analyses were conducted on Stata (StataCorp. 2021. Stata Statistical Software: Release 17. College Station, TX: StataCorp LLC).

## Results

Data on 11,655 participants were analysed within this study. A total of 5048 participants were excluded due to the following reasons: not returning the baseline ALSOP MEDICAL (*n* = 1547) and SOCIAL (*n* = 1593) questionnaires, study withdrawal (*n* = 667), incident dementia or cognitive decline before the PA exposure was captured (i.e. prior to return of the ALSOP questionnaire, *n* = 46) and had missing data for the exposure (*n* = 208) or confounders (*n* = 987) (Fig. 1). Compared to participants analysed within this study, those who were excluded were more likely to be females, received < 12 years of formal education, never drank alcohol, had lower annual income or have hypertension (Supplementary Table S1).

Participants’ baseline characteristics are shown by PA intensity in Table 1. Compared to participants who never/rarely engaged in PA or engaged in light PA, those who engaged in moderate or vigorous PA were more likely to be younger, male, obtained a higher level of formal education, have higher household income, live in more socioeconomically advantaged areas, consume alcohol, be an APOE4 carrier and be non-frail; however, they had lower mean BMI, were less likely to be current smokers, diabetic, hypertensive, or depressive symtpoms (Table 1).

**Table 1:** Baseline characteristics of 11,655 adults aged 70 years and over, according to physical activity intensity

Baseline characteristics	Total participants *N* = 11,655	Physical activity intensity			
		Rarely/Never *n* = 152	Light *n* = 3751	Moderate *n* = 5898	Vigorous *n* = 1854
Age, years mean ± SD	75.1 ± 4.2	76.7 ± 5.0	75.8 ± 4.6	74.8 ± 4.0	74.2 ± 3.6
Female, *n* (%)	6224 (53.4)	92 (60.5)	2384 (63.6)	2970 (50.4)	778 (42.0)
Education status, *n* (%)					
< 12 years	5490 (47.1)	93 (61.2)	1857 (49.5)	2787 (47.3)	753 (40.6)
≥ 12 years	6165 (52.9)	59 (38.8)	1894 (50.5)	3111 (52.7)	1101 (59.4)
Alcohol status, *n* (%)					
Current	9356 (80.3)	96 (63.2)	2869 (76.5)	4832 (81.9)	1559 (84.1)
Former	541 (4.6)	16 (10.5)	207 (5.5)	238 (4.0)	80 (4.4)
Never	1758 (15.1)	40 (26.3)	675 (18.0)	828 (14.1)	215 (11.5)
Smoking status, *n* (%)					
Current	326 (2.8)	6 (4.0)	128 (3.4)	161 (2.7)	31 (1.7)
Former	4822 (41.4)	66 (43.4)	1507 (40.2)	2469 (41.9)	780 (42.1)
Never	6507 (55.8)	80 (52.6)	2116 (56.4)	3268 (55.4)	1043 (56.2)
Living status, *n* (%)					
Alone	3443 (29.5)	72 (47.4)	1254 (33.4)	1613 (27.4)	504 (27.2)
Not alone	8212 (70.5)	80 (52.6)	2497 (66.6)	4285 (72.6)	1350 (72.8)
Income status (AUD per year), *n* (%)					
< $20,000	1767 (15.2)	45 (29.6)	638 (17.0)	845 (14.3)	239 (12.9)
$20,000–$49,999	6067 (52.0)	66 (43.4)	2078 (55.4)	3038 (51.5)	885 (47.7)
$50,000–$99,999	2084 (17.9)	17 (11.2)	538 (14.4)	1114 (18.9)	415 (22.4)
≥ $100,000	515 (4.4)	2 (1.3)	121 (3.2)	259 (4.4)	133 (7.2)
Prefer not to say	1222 (10.5)	22 (14.5)	376 (10.0)	642 (10.9)	182 (9.8)
IRSAD quintiles, *n* (%)					
1—least advantaged	1821 (15.6)	32 (21.1)	660 (17.6)	904 (15.3)	225 (12.1)
2	1962 (16.8)	25 (16.4)	650 (17.3)	980 (16.6)	307 (16.6)
3	2180 (18.7)	34 (22.3)	708 (18.9)	1108 (18.9)	330 (17.8)
4	2265 (19.4)	27 (17.8)	694 (18.5)	1146 (19.4)	398 (21.5)
5—most advantaged	3427 (29.4)	34 (22.4)	1039 (27.7)	1760 (29.8)	594 (32.0)
BMI, kg/m^2^ mean ± SD	27.9 ± 4.5	31.3 ± 6.4	29.0 ± 5.0	27.5 ± 4.1	26.7 ± 3.7
Diabetes mellitus, *n* (%)	1104 (9.5)	22 (14.5)	433 (11.5)	523 (8.9)	126 (6.8)
Hypertension, *n* (%)	8619 (74.0)	120 (79.0)	2964 (79.0)	4266 (72.3)	1269 (68.5)
Depression, *n* (%)	5999 (51.5)	90 (59.2)	2195 (58.5)	2897 (49.1)	817 (44.1)
Dyslipidaemia, *n* (%)	7808 (67.0)	97 (63.8)	2581 (68.8)	3932 (66.7)	1198 (64.6)
Frailty status, *n* (%)					
Not frail	7468 (64.1)	48 (31.6)	2027 (54.0)	4042 (68.5)	1351 (72.9)
Pre-frail/frail	4187 (35.9)	104 (68.4)	1724 (46.0)	1856 (31.5)	503 (27.1)
APOE4 carrier, *n* (%)	143 (1.2)	0 (0)	33 (0.9)	78 (1.3)	32 (1.7)

*SD* standard deviation, *IRSAD* Index of Relative Socioeconomic Advantage and Disadvantage; income status presented in Australian dollars, *APOE4* apolipoprotein E variant 4 allele

**Fig. 2:**
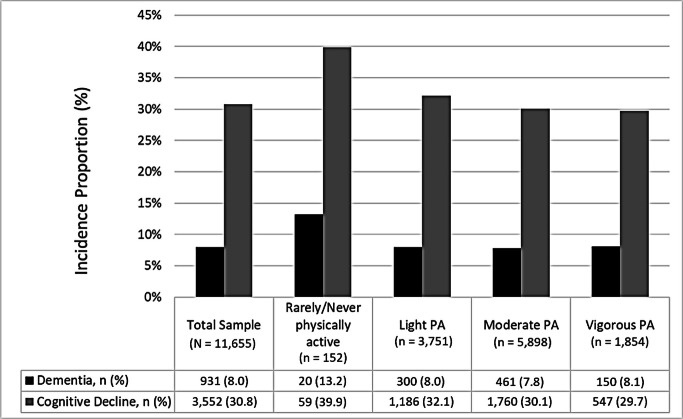
Cumulative incidence of dementia or cognitive decline in 11,655 community-dwelling older adults, overall and by intensity of physical activity. Numbers in the table represent the number and proportion of older adults who developed dementia and cognitive decline during a median 10.4-year follow-up

Over a median follow-up period of 10.4 years (25%, 9.3 years; 75%, 11.8 years), 931 (8.0%) participants developed incident dementia, and 3552 (30.8%) participants experienced cognitive decline. The cumulative incidence of dementia was highest among those who rarely/never engaged in PA (13.2%), then followed by vigorous PA (8.1%), light PA (8.0%) and moderate PA (7.8%) (Fig. 2). For cognitive decline, the highest cumulative incidence was observed in those who rarely/never engaged in PA (39.9%), followed by light PA (32.1%), moderate PA (30.1%) and vigorous PA (29.7%) (Fig. 2).

**Fig. 3:**
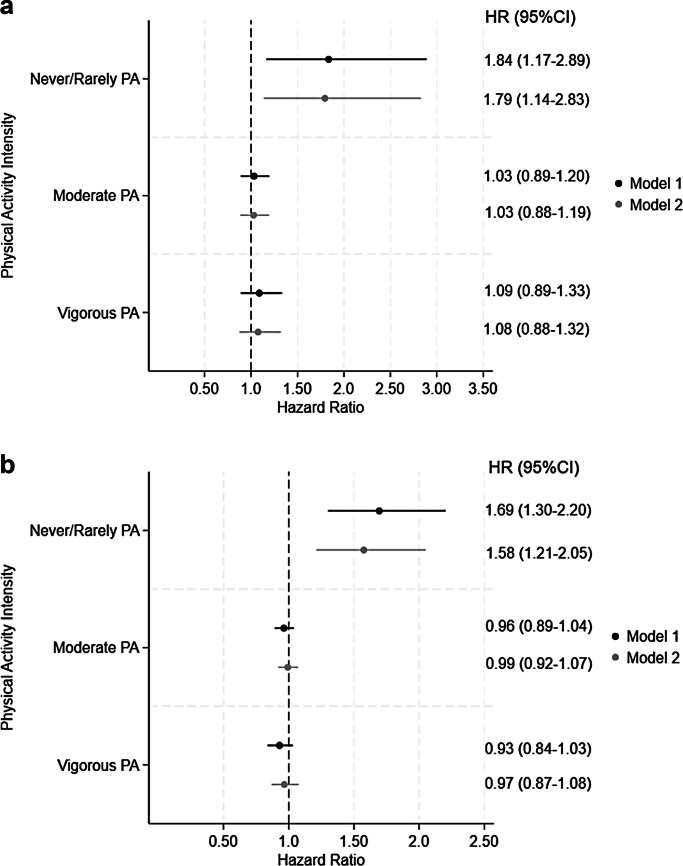
**a** The association between physical activity and dementia in 11,655 males and females aged 70 years and over, with light physical activity as the refence group: results of Cox regression analysis. Model 1: adjusted for age, sex, education, smoking, alcohol consumption, living status, income and IRSAD. Model 2: as Model 1 with additional adjustment for BMI, diabetes, hypertension, dyslipidaemia, depression and frailty. HR, hazard ratio; 95% CI, 95% confidence interval. **b** The association between physical activity and cognitive decline in 11,655 males and females aged 70 years and over, with light physical activity as the reference group: results of Cox regression analysis. Model 1: adjusted for age, sex, education, smoking, alcohol consumption, living status, income and IRSAD. Model 2: as Model 1 with additional adjustment for BMI, diabetes, hypertension, dyslipidaemia, depression and frailty. HR, hazard ratio; 95% CI, 95% confidence interval

In Model 2 of the Cox regression model, and compared to participants who reported engagement in light PA, the risk of developing dementia (HR 1.79, 95% CI 1.14–2.83) or experiencing cognitive decline (HR 1.58, 95% CI 1.21–2.05) was higher in older adults reporting to rarely/never engage in PA. The risk of dementia or experiencing cognitive decline was similar between older adults reporting engagement in light PA and those reporting engagement in moderate or vigorous PA (Fig. 3a and b).

After performing sensitivity analysis, using participants who never/rarely engage in PA as the reference category and adjustments for putative confounders, engaging in any PA intensity had a lower risk of developing incident dementia (HR 0.57, 95% CI 0.36–0.89) or cognitive decline (HR 0.63, 95% CI 0.49–0.82) (Supplementary Figure S3a and S3b).

Results of other sensitivity analyses are provided in Supplementary tables and produced similar results to the main analysis: with participants excluded if they developed dementia or cognitive decline within the first year from study enrolment (Supplementary Table S2), with additional adjustments for self-reported PA during middle-age and APOE4 carrier status (Supplementary Table S3), and after applying multiple imputation for all missing covariates and exposures (*n* = 3191) (Supplementary Table S4).

## Discussion

In this longitudinal study of 11,655 adults aged 70 years and older, we observed that participants who never/rarely engaged in PA had a 79% higher risk of developing dementia and 58% higher risk of experiencing cognitive decline when compared to those engaging in light PA. When those who never/rarely engaged in PA were compared to any PA intensity, a 53% lower risk was observed for dementia and 37% lower risk for cognitive decline. This suggests that any intensity of PA may be beneficial for older adults in delaying the onset of dementia and cognitive decline, highlighting the importance of avoiding physical inactivity.

Our study findings align with the few prior studies conducted, albeit in differing cohort demographics [8, 9, 29]. The Neurological Disorders in Central Spain Study (NEDICES) included 3105 Spanish older adults with baseline mild cognitive decline, collected self-reported PA intensities and found a 47% lower risk of dementia for those who engaged in light PA, 55% for moderate and 71% for high PA intensity, when compared to being sedentary [8]. Yet, the results of this study have limited context to initially healthy older adults (without cognitive decline) within Australia, where PA patterns may be different to those with mild cognitive impairment [8]. The CHARLS study included 5206 middle-aged and older adults and examined the association of light, moderate and vigorous PA intensities with mental intactness, episodic memory and overall cognition using a prediction model and found that both light (*β* = 0.271, 95% CI 0.178–0.912) and moderate PA (*β* = 0.486, 95% CI 0.514–1.224) were associated with improved cognitive scores [9]. However, the CHARLS study did not stratify analyses by middle and older-aged participants, and considering that older adults may have different risk profiles and PA patterns compared to middle-aged adults, the protective effect of PA for dementia and cognitive decline may be over/underestimated [9]. Lastly, the Whitehall II cohort study, which included > 90,000 older adults in the UK and employed accelerometers to objectively measure PA, reported a 69% reduction in dementia risk among individuals engaging in ≥ 140 min of moderate-to-vigorous PA per week, compared to those with no engagement in PA [31]. However, the Whitehall II study did not investigate if light PA offered a similar protective effect against dementia and cognitive decline. Light PA is especially important, as not all older adults are able to engage in higher intensities of PA and capitalise on the protective effects. Our study extends existing evidence by evaluating the relationship between light, moderate and vigorous PA intensities and dementia and cognitive decline in Australian community-dwelling older adults, demonstrating similar protective associations across all intensities and adding granularity to prior research.

While previous research has often promoted vigorous PA as more beneficial, these studies typically relied on a younger sample population (age 55 years or older), binary comparisons (yes vs no, high vs low or moderate-to-vigorous vs light), smaller sample sizes (*N* < 5000) and shorter follow-up periods (< 10 years), limiting their ability to capture long-term effects and the nuanced impact of PA intensity [3, 4, 32]. In contrast, our study of 11,655 participants with a median 10.4-year follow-up period revealed a U-shaped relationship (similar to the Mendelian randomisation trial) when PA is measured across varying intensities, and was confirmed through our sensitivity analysis. One plausible explanation is that engaging in vigorous PA intensity may lead to negative effects on cognition, such as minor impairments in memory or reduced cognitive resilience, that are unlikely to be detected in short-term studies [10, 11, 33]. The Mendelian randomisation trial that used genetic proxies to predict moderate-vigorous PA and risk of developing dementia suggested that this was a result of increased reactive oxygen species, leading to oxidative stress and neuronal damage [10]. Yet, prediction models only estimate associations and may not accurately reflect true PA intensities and account for confounding influences across the lifespan, which may incur misclassification or measurement errors. Roy et al. proposed a similar point, where high-intensity PA may increase fatigue and heart rates and decrease brain activity [11]. However, Roy’s study was only conducted among 20 children aged 8–17 years, which may have limited the power of their analysis, their ability to account for modifying risk factors such as education and their results may not be generalisable to an older adult population with varying risk factors [11]. Our findings, based on direct observational data (measured directly in participants through assessments and questionnaires, rather than inferred from genetic proxies or prediction models) in older adults and long-term follow-up in a large cohort, suggest that even light-intensity PA may be sufficient to reduce the risk of dementia and cognitive decline during older age.

The mechanisms underlying the observed associations may be multifactorial. PA is known to enhance cerebral blood flow [34], reduce inflammation [33] and promote neurogenesis [35], particularly in regions such as the hippocampus that are critical for memory and learning [34, 35]. In older adults, light-intensity PA may be sufficient to activate these foundational neuroprotective pathways while remaining physiologically tolerable and sustainable [36]. In contrast, engaging in higher-intensity activity may not always confer additional benefit in later life. As antioxidant defences decline with age, increasing the intensity/duration of PA could induce physiological stress, such as elevated cortisol, suppressed immune system, oxidative stress or cardiovascular strain, which could negatively affect neural plasticity and cognition [36]. These adverse effects may offset the expected neuroprotective benefits of vigorous PA, particularly in older adults with reduced resilience or comorbidities [36].

### Implications for clinical practice, health policy and future research

Our results indicate that engaging in light-intensity PA may offer comparable protective benefits against incident dementia and cognitive decline as moderate or vigorous PA. Our findings suggest that clinicians should encourage any level of PA, rather than focusing solely on moderate or vigorous exercise, which may be unattainable for adults aged 70 and above. In practice, this means that healthcare providers can reassure older adults that engaging in light activities, such as walking, stretching, or household chores, still offers cognitive benefits [12]. Embedding PA promotion into routine clinical encounters, preventive health assessments and care planning for older adults may therefore be an effective strategy to preserve cognitive function and quality-of-life [37]. Promotion and interventions should also be implemented early, targeting not only older adults but young and middle-aged adults, as lifelong engagement in PA may infer a greater protective effect against cognitive decline than starting PA engagement later on in life [38]. Lastly, PA may also act as a marker of a broader health-promoting lifestyle. Unmeasured factors such as diet quality, sleep health, social engagement and cognitive or mental stimulation, none of which were assessed in the present study, are associated with both PA behaviours and cognitive outcomes and therefore may contribute to the observed associations [39]. Maintaining healthier diets, better sleep routines and greater social or cognitive engagement are known to contribute to cognitive health preservation and can also reinforce cognitive maintenance [39]. Future research should investigate how light PA (using objective measures) interacts with other modifiable risk factors, such as diet and obstructive sleep apnoea to influence cognitive trajectories [39].

### Strengths and limitations

To the best of our knowledge, this is the first large (11,655) longitudinal cohort study in community-dwelling older Australian adults (median follow-up of 10.4 years) to examine the relationship between varying intensities of PA and dementia and cognitive decline. Notably, we adjusted for APOE carrier status, a genetic risk factor for dementia which is rarely accounted for in comparable studies, and used expert adjudicated dementia outcomes, thereby enhancing the robustness of our findings.

Despite its strengths, this study has several limitations. Our cohort was predominantly Caucasian (97%) and largely comprised of individuals from higher socio-economic backgrounds, limiting the generalisability of our findings. Differences in ethnicities and cultural variations may shape both PA patterns and dementia risk, flagging the need for studies in more ethnically and socioeconomically diverse populations to assess the generalisability of these findings. PA was self-reported, which may have introduced recall bias and measurement error leading to exposure misclassification, due to individual differences in the perception of activity intensity (i.e., what constitutes light, moderate or vigorous activity) [40]. To minimise the possibility of misclassification bias, the ALSOP PA questionnaire included several examples of activities within each intensity category to assist participants in selecting the most appropriate intensity of PA. Moreover, self-reported PA measures remain feasible for large-scale epidemiological studies and have demonstrated reasonable validity for categorising individuals by relative activity level, although with limited precision for distinguishing exact intensity gradients, suggesting that true dose–response relationships may be underestimated [40]. As PA was assessed prior to dementia diagnosis, any misclassification is likely to be non-differential and would therefore be expected to attenuate associations toward the null, which may partially explain the observed U-shaped pattern at higher activity intensities [41]. We did not have information on the type, frequency, and duration of PA, factors that may differentially influence dementia and cognitive decline risk [3] We also did not account for confounders such as diet and poor sleeping patterns, which may lead to residual confounding bias [39]. Although a threshold or U-shaped pattern was observed, this finding should be interpreted with caution. The attenuation of benefit at higher intensities may partly reflect non-causal explanations, including reverse causality (despite conducting a sensitivity analysis), health selection, and functional limitations that influence both PA intensity and cognitive outcomes in older adults.

## Conclusion

This study suggests that engagement at any PA intensity, including light-intensity PA, is associated with a lower risk of dementia and cognitive decline in older adults. Although causal inferences cannot be drawn from this cohort study, the findings underscore the potential importance for clinicians to promote achievable activities for older individuals, such as walking or household tasks, that are sustainable and may support cognitive health and healthy aging.

## Supplementary information

Below is the link to the electronic supplementary material.

ESM 1 (DOCX 714 KB)

## Data Availability

Our research data includes sensitive or confidential information such as patient data that will be unavailable for public access. However, a de-identified dataset is available, upon reasonable form ASPREE investigators (https://ams.aspree.org/).
