# Supplementary material for: Association of physical activity with incident dementia and cognitive decline among Australian older adults
Source: GeroScience. 2026 Mar 4;48(3):3415–28. doi: 10.1007/s11357-026-02179-x (PMC13356129; doi:10.1007/s11357-026-02179-x)
Supplement: Supplementary file 1 — (DOCX 714 KB) [file 11357_2026_2179_MOESM1_ESM.docx]

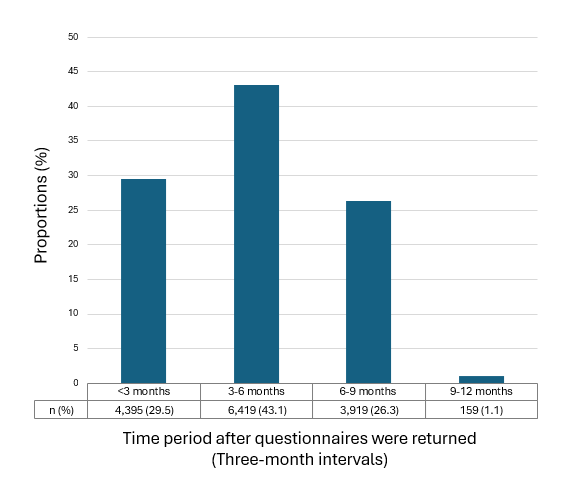


**Supplementary Figure S1:** Number and proportion of participants that returned the baseline questionnaires at each time period


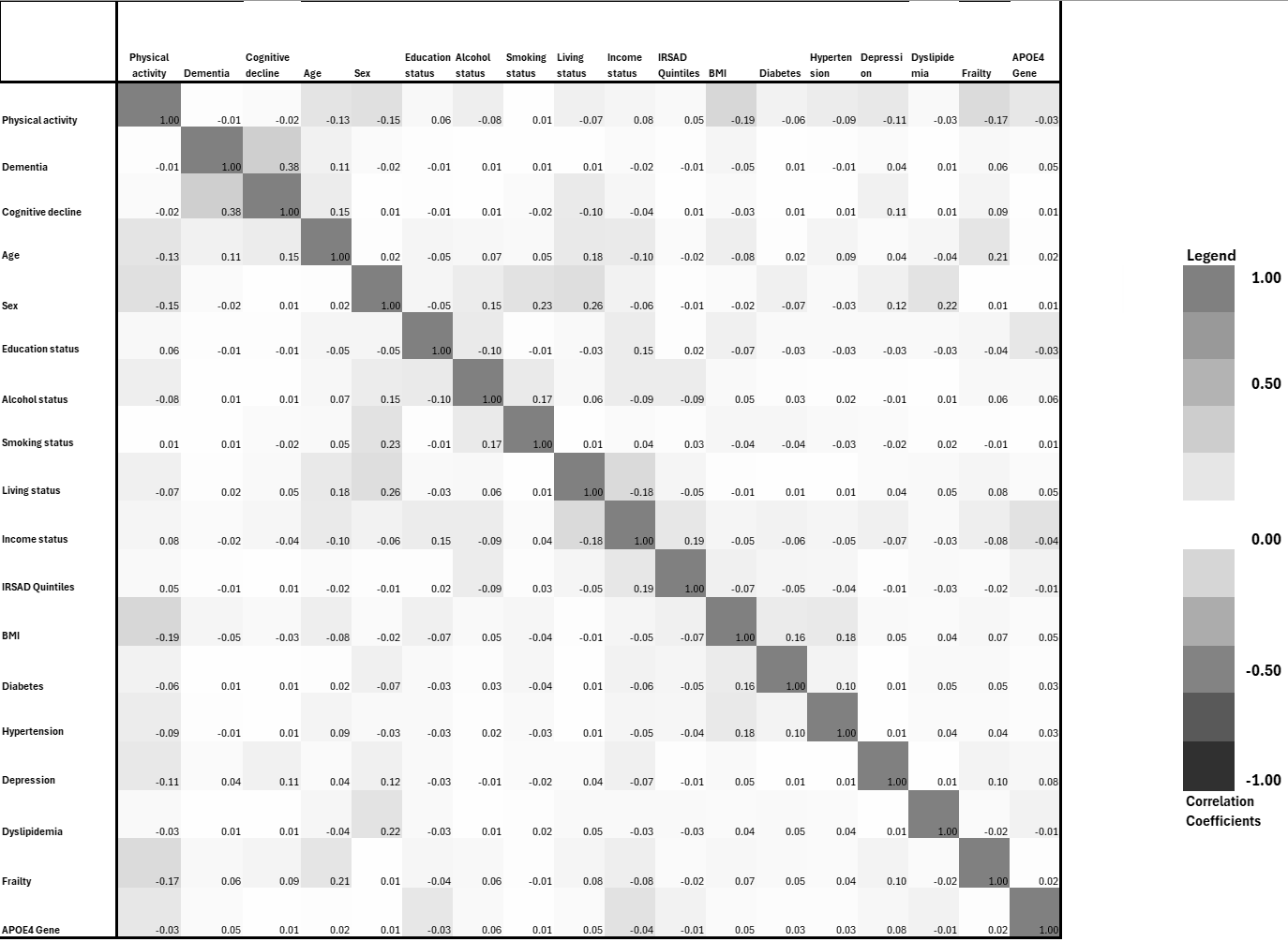


**Supplementary Figure S2:** Spearman correlation test of physical activity with dementia and cognitive decline and covariates depicted using a heatwave figure


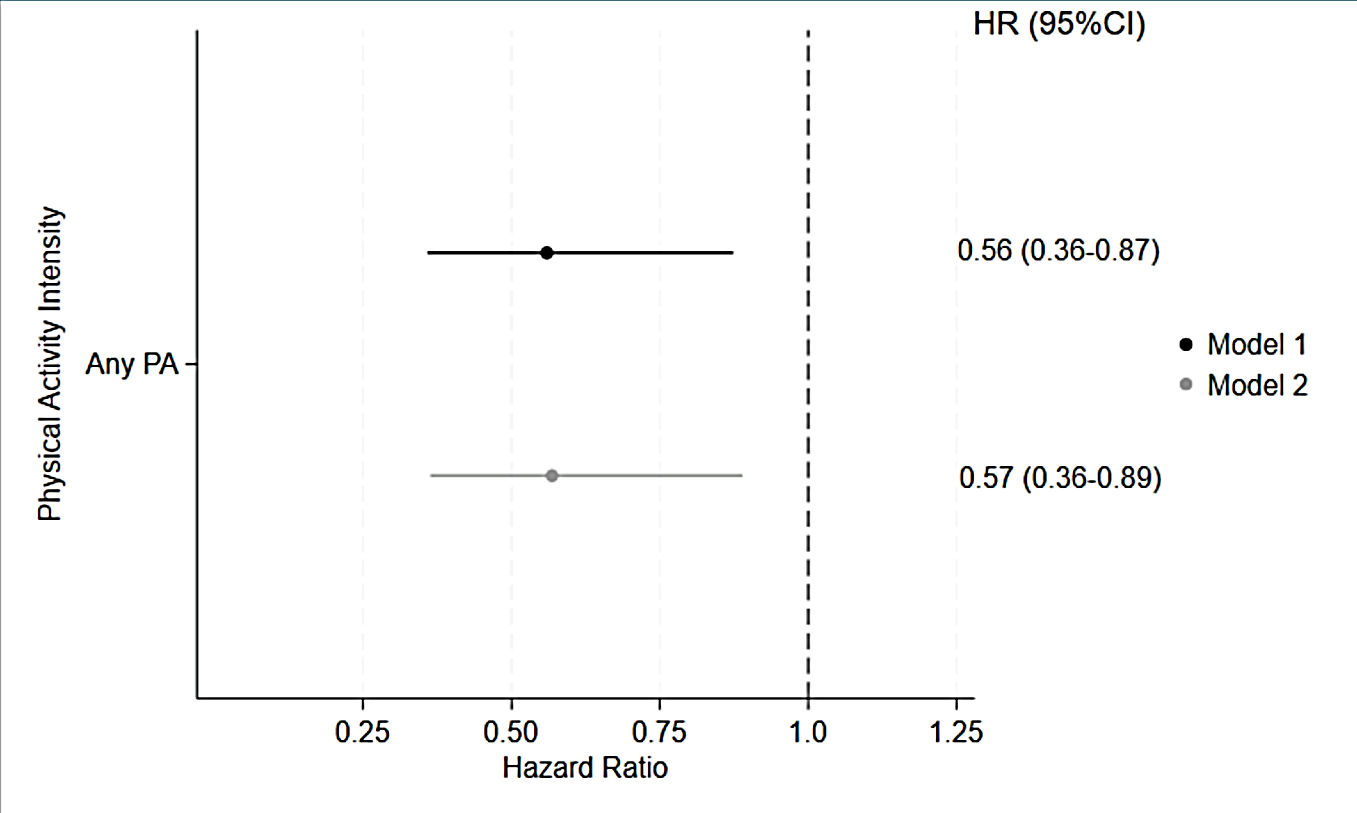


**Supplementary Figure S3a:** Sensitivity analysis using never/rarely engaged in physical activity as the reference category and combining all other PA intensities together (‘any’ PA), on the association between physical activity and dementia in 11,655 males and females aged 70 years and over: results of a Cox regression analysis.

**Model 1:** adjusted for age, sex, education, smoking, alcohol consumption, living status, income, and IRSAD. **Model 2:** as Model 1 with additional adjustment for BMI, diabetes, hypertension, dyslipidaemia, depression, and frailty. HR – Hazard ratio, 95% CI – 95% confidence interval.


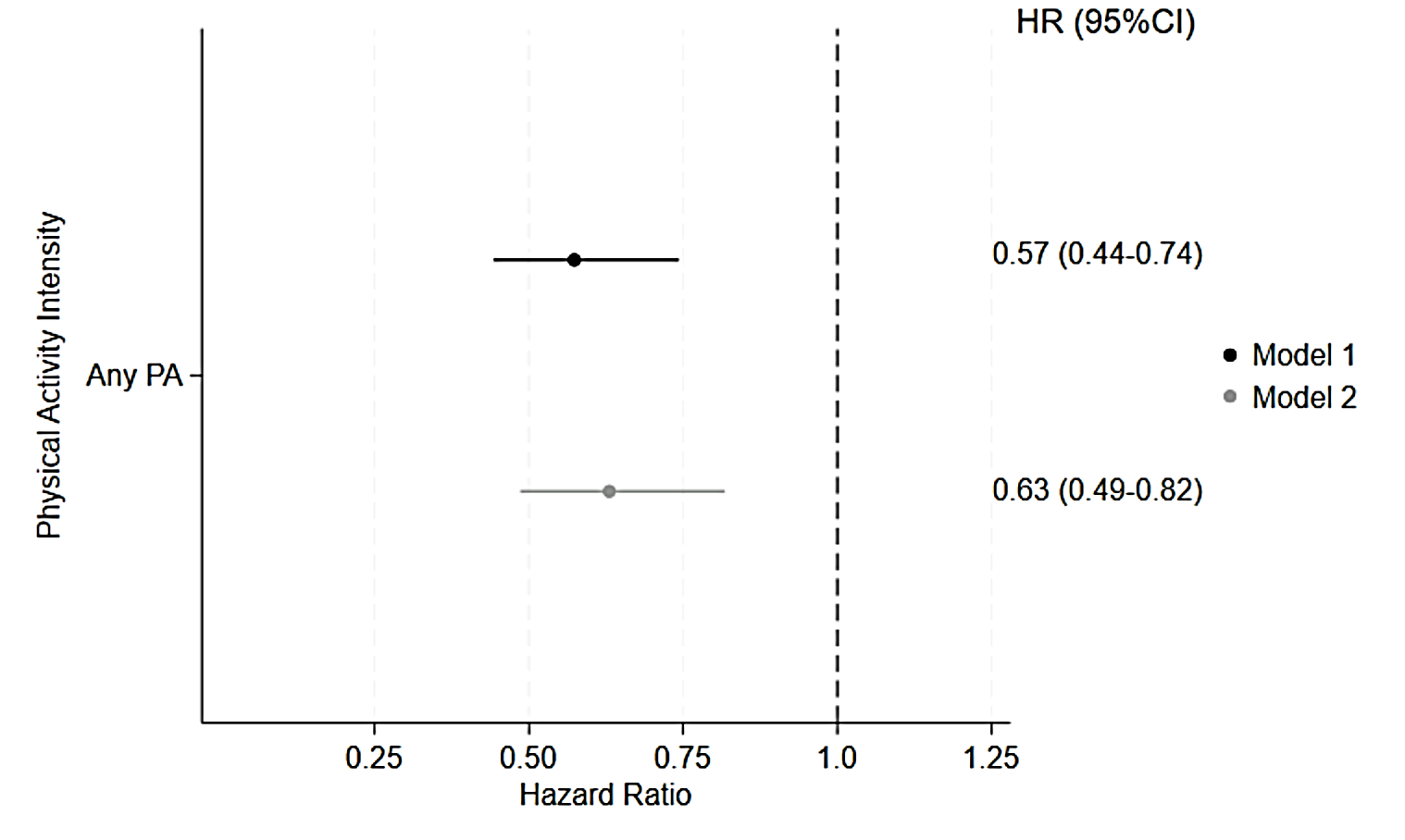


**Supplementary Figure S3b:** Sensitivity analysis using never/rarely engaged in physical activity as the reference category and combining all other PA intensities together (‘any’ PA), on the association between physical activity and cognitive decline in 11,655 males and females aged 70 years and over: results of a Cox regression analysis.

**Model 1:** adjusted for age, sex, education, smoking, alcohol consumption, living status, income, and IRSAD. **Model 2:** as Model 1 with additional adjustment for BMI, diabetes, hypertension, dyslipidaemia, depression, and frailty. HR – Hazard ratio, 95% CI – 95% confidence interval.

**Supplementary Table S1:** Comparison of participants included within this study and participants excluded for missing covariates

| **Baseline characteristics** | **Participants not missing data**  **n= 11,655** | **Participants missing data**  **n = 3,237** |
| --- | --- | --- |
|  |  |  |
| Age, years mean ± SD | 75.1 ± 4.2 | 75.5 ± 4.8 |
| Female, n (%) | 6,224 (53.4) | 2,049 (63.3) |
| Education status, n (%)  < 12 years  ≥ 12 years | 5,490 (47.1)  6,165 (52.9) | 1,819 (56.2)  1,418 (43.8) |
| Alcohol status, n (%)  Current  Former  Never | 9,356 (80.3)  541 (4.6)  1,758 (15.1) | 2,480 (76.6)  110 (3.4)  647 (20.0) |
| Smoking status, n (%)  Current  Former  Never | 326 (2.8)  4,822 (41.4)  6,507 (55.8) | 117 (3.6)  1,279 (39.5)  1,841 (56.9) |
| Living status, n (%)  Alone  Not alone | 3,443 (29.5)  8,212 (70.5) | 800 (31.1)  1,772 (68.9) |
| Income status (AUD per year), n (%)  < $20,000  $20,000-$49,999  $50,000- $99,999  ≥ $100,000  Prefer not to say | 1,767 (15.2)  6,067 (52.0)  2,084 (17.9)  515 (4.4)  1,222 (10.5) | 508 (21.5)  1,223 (51.8)  293 (12.4)  73 (3.1)  264 (11.2) |
| IRSAD quintiles, n (%)  1 -Least advantaged  2  3  4  5 – Most advantaged | 1,821 (15.6)  1,962 (16.8)  2,180 (18.7)  2,265 (19.4)  3,427 (29.4) | 442 (18.7)  423 (17.9)  420 (17.8)  427 (18.1)  649 (27.5) |
| BMI, kg/m^2^ mean ± SD | 27.9 ± 4.5 | 28.8 ± 5.0 |
| Diabetes mellitus, n (%) | 1,104 (9.5) | 330 (10.2) |
| Hypertension, n (%) | 8,619 (74.0) | 2,518 (77.8) |
| Depression, n (%) | 5,999 (51.5) | 1,725 (53.3) |
| Dyslipidaemia, n (%) | 7,808 (67.0) | 2,246 (69.4) |
| Frailty status, n (%)  Not Frail  Prefrail/Frail | 7,468 (64.1)  4,187 (35.9) | 2,013 (62.2)  1,224 (37.8) |
| APOE4 carrier, n (%) | 143 (1.2) | 62 (1.9) |

SD – standard deviation, IRSAD – Index of Relative Socioeconomic Advantage and Disadvantage; Income status presented in Australian dollars, APOE4 - apolipoprotein E variant 4 allele

**Supplementary Table S2:** Sensitivity analysis on the association of physical activity with dementia and cognitive decline in 11,577 males and females aged 70 years and over, excluding n=78 participants who developed the dementia or cognitive decline within the first year.

| Physical activity engagement: | Events, n (%) | Model 1  HR (95%CI) | Model 2  HR (95%CI) |
| --- | --- | --- | --- |
| Dementia | | | |
| Rarely/never | 17 (11.3) | 1.84 (1.15-2.93) | 1.80 (1.13-2.87) |
| Light (ref) | 272 (7.3) | - | - |
| Moderate | 431 (7.3) | 1.06 (0.92-1.24) | 1.06 (0.91-1.24) |
| Vigorous | 141 (7.6) | 1.12 (0.91-1.38) | 1.11 (0.90-1.36) |
| Cognitive Decline | | | |
| Rarely/never | 43 (28.7) | 1.71 (1.31-2.23) | 1.59 (1.21-2.08) |
| Light (ref) | 1,104 (29.7) | - | - |
| Moderate | 1,665 (28.4) | 0.97 (0.90-1.05) | 1.01 (0.93-1.08) |
| Vigorous | 513 (27.8) | 0.94 (0.84-1.04) | 0.97 (0.87-1.08) |

**Model 1:** adjusted for age, sex, education, smoking, alcohol consumption, living status, income, and IRSAD. **Model 2**: adjusted for all previous covariates and BMI, diabetes, hypertension, depression, dyslipidaemia and frailty. HR – Hazard ratio, 95% CI – 95% confidence interval.

**Supplementary Table A.3:** Sensitivity analysis on the association of physical activity with dementia and cognitive decline in 11,655 males and females aged 70 years and over, with additional adjustment for APOE4 carrier status and physical activity engagement during middle-age.

| Physical activity engagement: | Events, n (%) | Model 1  HR (95%CI) | Model 2  HR (95%CI) |
| --- | --- | --- | --- |
| Dementia | | | |
| Rarely/never | 20 (13.2) | 1.80 (1.13-2.87) | 1.77 (1.11-2.82) |
| Light (ref) | 300 (8.0) | - | - |
| Moderate | 461 (7.8) | 1.04 (0.89-1.21) | 1.02 (0.88-1.19) |
| Vigorous | 150 (8.1) | 1.09 (0.89-1.33) | 1.05 (0.86-1.30) |
| Cognitive Decline | | | |
| Rarely/never | 59 (39.9) | 1.67 (1.27-2.20) | 1.54 (1.16-2.05) |
| Light (ref) | 1,186 (32.1) | - | - |
| Moderate | 1,760 (30.1) | 0.97 (0.90-1.05) | 0.98 (0.91-1.07) |
| Vigorous | 547 (29.7) | 0.93 (0.84-1.04) | 0.94 (0.84-1.06) |

**Model 1:** adjusted for age, sex, education, smoking, alcohol consumption, living status, income, and IRSAD. **Model 2**: adjusted for all previous covariates and BMI, diabetes, hypertension, depression, dyslipidaemia, frailty, APOE4 carrier and PA during middle-age. HR – Hazard ratio, 95% CI – 95% confidence interval.

**Supplementary Table S4:** Cox regression analysis using multiple imputations to impute for missing data on the association of physical activity with dementia and cognitive decline in 14,846 males and females aged 70 years and over.

| Physical activity engagement: | Events, n (%) | Model 1  HR (95%CI) | Model 2  HR (95%CI) |
| --- | --- | --- | --- |
| Dementia | | | |
| Rarely/never | 32 (14.6) | 1.75 (1.08-2.44) | 1.73 (1.07-2.40) |
| Light (ref) | 409 (8.5) | - | - |
| Moderate | 624 (8.3) | 1.03 (0.92-1.21) | 1.04 (0.93-1.21) |
| Vigorous | 204 (8.7) | 1.05 (0.92-1.21) | 1.07 (0.94-1.22) |
| Cognitive Decline | | | |
| Rarely/never | 88 (40.9) | 1.44 (1.14-1.88) | 1.42 (1.12-1.86) |
| Light (ref) | 1,591 (33.1) | - | - |
| Moderate | 2,333 (31.0) | 0.98 (0.90-1.03) | 0.99 (0.93-1.04) |
| Vigorous | 712 (30.4) | 0.94 (0.85-1.05) | 0.96 (0.85-1.07) |

**Model 1:** adjusted for age, sex, education, smoking, alcohol consumption, living status, income, and IRSAD. **Model 2**: adjusted for all previous covariates and BMI, diabetes, hypertension, depression, dyslipidaemia and frailty. HR – Hazard ratio, 95% CI – 95% confidence interval.

**Supplementary Methods**

Multiple Imputation:

The choice to utilise multiple imputations was mainly due to a large number of missing data for certain covariates/ PA exposure having that would have a high impact on the outcomes. For example, income information was missing for 692 participants, followed by education of 192 participants and physical activity with 208 participants.

Missing covariate data were addressed using multiple imputation by chained equations (MICE) under a missing at random (MAR) assumption. A total of 20 imputations were generated to ensure the best approximation of the proportion of incomplete cases (~21%).

The imputation model included all covariates used in the main analyses, including: age, sex, education, smoking, alcohol consumption, living status, income, IRSAD, BMI, diabetes, hypertension, depression, dyslipidaemia and frailty. The MICE model also included missing physical activity (exposure) data. BMI and age (continuous variables) were imputed using predictive mean matching to preserve the observed data distribution while categorical variables were imputed using ordinal logistic or multinomial logistic regression models. For time-to-event analyses, the event indicator (dementia or cognitive decline) and the Nelson–Aalen cumulative hazard estimate were included in the imputation model. Each imputed dataset was analysed using Cox proportional hazards regression, and the resulting hazard ratios (HRs) and 95% confidence intervals (CIs) were combined across imputations using Rubin’s rules.
